# Supplementary material for: Longstanding smoking associated with frontal brain lobe atrophy: a 32-year follow-up study in women
Source: BMJ Open. 2023 Oct 6;13(10):e072803. doi: 10.1136/bmjopen-2023-072803 (PMC10565256; doi:10.1136/bmjopen-2023-072803)
Supplement: Supplementary data [file bmjopen-2023-072803supp001.pdf]

Additional file Correlation matrix presenting Pearson correlation coefficient (95% CI) between the five outcome variables

	Temporal lobe atrophy	Frontal lobe atrophy	Parietal lobe atrophy	Occipital lobe atrophy
Frontal lobe atrophy	0.49 (0.41-0.57) ***			
Parietal lobe atrophy	0.52 (0.44-0.59) ***	0.55 (0.47-0.62) ***		
Occipital lobe atrophy	0.54 (0.46-0.61) ***	0.48 (0.39-0.55) ***	0.70 (0.64-0.75) ***	
White matter lesions	0.23 (0.13-0.32) ***	0.15 (0.05-0.25) **	0.15 (0.05-0.25) **	0.21 (0.12-0.31) ***

The Pearson correlation coefficient (95% CI), *** = p value <0.001, ** = p value <0.01
