# Supplementary material for: Longstanding smoking associated with frontal brain lobe atrophy: a 32-year follow-up study in women
Source: BMJ Open. 2023 Oct 6;13(10):e072803. doi: 10.1136/bmjopen-2023-072803 (PMC10565256; doi:10.1136/bmjopen-2023-072803)
Supplement: Author's manuscript [file bmjopen-2023-072803.draft_revisions.pdf]

BMJ Open

BMJ Open is committed to open peer review. As part of this commitment we make the peer review history of every article we publish publicly available.

When an article is published we post the peer reviewers' comments and the authors' responses online. We also post the versions of the paper that were used during peer review. These are the versions that the peer review comments apply to.

The versions of the paper that follow are the versions that were submitted during the peer review process. They are not the versions of record or the final published versions. They should not be cited or distributed as the published version of this manuscript.

BMJ Open is an open access journal and the full, final, typeset and author-corrected version of record of the manuscript is available on our site with no access controls, subscription charges or pay-per-view fees (http://bmjopen.bmj.com).

If you have any questions on BMJ Open's open peer review process please email info.bmjopen@bmj.com

BMJ Open

Longstanding smoking associated with frontal brain lobe atrophy: A 32-year follow-up study

Journal:	*BMJ Open*
Manuscript ID	bmjopen-2023-072803
Article Type:	Original research
Date Submitted by the Author:	24-Feb-2023

Complete List of Authors:	Johansson, Lena; Goteborgs universitet Sahlgrenska Akademin, Institute of Neuroscience and Physiology<br>Guo, Xinxin; University of Gothenburg, Department of Psychiatry and Neurochemistry<br>Sacuiu, S; Goteborgs Universitet,<br>Fässberg, Madeleine; University of Gothenburg Institute of Neuroscience and Physiology, Department of Psychiatry and Neurochemistry<br>Kern, Silke; University of Gothenburg Institute of Neuroscience and Physiology, Department of Psychiatry and Neurochemistry<br>Zettergren, Anna; University of Gothenburg Institute of Neuroscience and Physiology, Department of Psychiatry and Neurochemistry<br>Skoog, Ingmar; University of Gothenburg Institute of Neuroscience and Physiology, Department of Psychiatry and Neurochemistry

Keywords:	EPIDEMIOLOGIC STUDIES, NEUROPATHOLOGY, Old age psychiatry < PSYCHIATRY

SCHOLARONE™
Manuscripts

I, the Submitting Author has the right to grant and does grant on behalf of all authors of the Work (as defined in the below author licence), an exclusive licence and/or a non-exclusive licence for contributions from authors who are: i) UK Crown employees; ii) where BMJ has agreed a CC-BY licence shall apply, and/or iii) in accordance with the terms applicable for US Federal Government officers or employees acting as part of their official duties; on a worldwide, perpetual, irrevocable, royalty-free basis to BMJ Publishing Group Ltd ("BMJ") its licensees and where the relevant Journal is co-owned by BMJ to the co-owners of the Journal, to publish the Work in this journal and any other BMJ products and to exploit all rights, as set out in our licence.

The Submitting Author accepts and understands that any supply made under these terms is made by BMJ to the Submitting Author unless you are acting as an employee on behalf of your employer or a postgraduate student of an affiliated institution which is paying any applicable article publishing charge ("APC") for Open Access articles. Where the Submitting Author wishes to make the Work available on an Open Access basis (and intends to pay the relevant APC), the terms of reuse of such Open Access shall be governed by a Creative Commons licence – details of these licences and which Creative Commons licence will apply to this Work are set out in our licence referred to above.

Other than as permitted in any relevant BMJ Author's Self Archiving Policies, I confirm this Work has not been accepted for publication elsewhere, is not being considered for publication elsewhere and does not duplicate material already published. I confirm all authors consent to publication of this Work and authorise the granting of this licence.

For peer review only

Longstanding smoking associated with frontal brain lobe atrophy: A 32-year follow-up study in women

Lena Johansson [ab] Xinxin Guo [a], Simona Sacuiu [a], Madeleine Mellqvist Fässberg [a], Silke Kern [a], Anna Zettergren [a], Ingmar Skoog [a]

Affiliations

[a] Department of Psychiatry and Neurochemistry, Sahlgrenska Academy, Centre for Ageing and Health (AgeCap) at the University of Gothenburg, Sweden

[b] Region Västra Götaland, Sahlgrenska University Hospital, Addiction Clinic, Gothenburg, Sweden

Address for correspondence: Lena Johansson, University of Gothenburg and Sahlgrenska University Hospital, Institute of Neuroscience and Physiology, Department of Psychiatry and Neurochemistry, Unit of Psychiatric Epidemiology, Wallinsgatan 6, 431 41 Mölndal, Sweden. Tel: +46 31 343 86 47 Fax: +46 31 776 04 03 E-mail: lena.johansson@neuro.gu.se

Abbreviations: BMI: body mass index, CCVD: cardio- and cerebrovascular diseases, CI: confidence intervals, CT: computerized tomography, MRI: magnetic resonance imaging, OR: odds ratios, VBM: voxel-based morphometry

For peer review only

ABSTRACT

Objective To examine the association between midlife tobacco smoking and late-life brain atrophy and white matter lesions.

Methods The study includes 369 women from the Prospective Population Study of Women in Gothenburg, Sweden. Cigarette smoking was reported in baseline 1968 (mean age = 44) and in two follow-ups, in 1974-75 and 1980-81. Brain computerized tomography (CT) was conducted 32 years after baseline examination (mean age = 76) and measured cortical atrophy and white matter lesions. Logistic regression models estimated associations between midlife smoking and late-life brain lesions. The final analyses were adjusted for alcohol consumption, vascular disorders, and several other potential confounders.

Results Reported smoking in 1968-69 (adjusted OR 1.85; 95% CI 1.12-3.04), in 1974-75 (OR 2.37; 95% CI 1.39-4.04), and in 1980-81 (OR 2.47; 95% CI 1.41-4.33) were associated with late-life frontal lobe atrophy (2000-01). The strongest association was observed in women who reported smoking at all three midlife examinations (OR 2.63; 95% CI 1.44-4.78) and in those with more frequent alcohol consumption (OR 6.02; 95% CI 1.74-20.84). Smoking in 1980-81 was also associated with late-life parietal lobe atrophy (OR 1.99 95%; CI 1.10-3.58). There were no associations between smoking and atrophy in the temporal or occipital lobe, or with white matter lesions.

Conclusion Longstanding tobacco smoking was mainly associated with atrophy in frontal lobe cortex. A long-term stimulation of nicotine receptors in the frontal neural pathway might be harmful for targeted brain cell.

- **What is already known on this topic** – Short-term effects of tobacco on the body and brain are quite well investigated.

For peer review only

- **What this study adds** – This study found that midlife tobacco smoking was associated with late-life frontal brain atrophy. No similar population-based study has earlier found this pattern.
- **How this study might affect research, practice, or policy** – The study adds information to smokers and health care professionals about eventual risks of tobacco use and propose increased research about brain damaging effects of longstanding smoking.

For peer review only

INTRODUCTION

Cigarette smoking continues to be one of the most common addictive habits globally and is the single greatest cause of preventable death.[1] Tobacco consists of thousands of harmful compounds, of which many are directly toxic to the brain and the central nervous system (CNS).[2] The short-term effect on the brain is rather well known, but less research has been done regarding long-term consequences.

Brain atrophy increase with age, often with an unidentified aetiology.[3, 4] Cortical atrophy is due to advanced loss of neurons in gray matter areas, expressed generally in entire cortex, or partial in specific areas. Gray matter neurodegeneration can be a result of for example proteopathies, inflammatory processes, or oxidative stress.[4] Sub-cortical lesions are more often related to cerebrovascular damages, such as small-vessel disease or brain infarcts, and are related to widespread damages on the cell axons in white matter areas.[5] The consequences of extended cortical and sub-cortical shrinkage can be seen as cognitive and functional impairment.[4]

A number of genetic and life-style factors have been associated with structural brain changes.[6-10] Tobacco use poses a risk to the brain due to the exposure of toxic chemicals, but only a few studies have examined long-term associations between cigarette smoking and late-life brain lesions.[11, 12, 13] The most addictive and intoxicating ingredients in tobacco is nicotine, an alkaloid that mainly binds to acetylcholine receptors in CNS,[14] and interact with the dopaminergic reward system.[15, 2] Chronic tobacco smoking increases the amount of nicotine receptors in the brain and changes the structure and function of involved neurons.[2] Nicotine has also negative vascular effects in the brain, due to arteriolosclerosis and heightening of blood pressure.[2, 16]

For peer review only

In this study, we analyse the relation between midlife tobacco smoking and presence of late-life gray matter atrophy and white matter lesions on computerized tomography (CT), in a population-based sample of 369 women followed over 32 years. We have an extra focus on alcohol consumption, given its high co-morbidity with smoking and emerging findings regarding their interactions.[17] The study adjusts for a number of important covariates, e.g., vascular risk factors.

METHODS

Study population

The study is part of the Prospective Population Study of Women in Gothenburg which was initiated in 1968 with a comprehensive examination of 1462 women (participation rate 90%) born in 1908, 1914, 1918, 1922 and 1930.[18, 19] The individuals were systematically sampled from the Swedish Population Register, based on specific birth dates, to yield a representative sample. Follow-ups were performed in 1974-75, 1980-81, 1992-93, and 2000-01, with participation rates among survivors of 91%, 83%, 70%, and 71%, respectively. At the examination in 2000-01, all participants (n=684) were invited for brain CT-scan. Participants in the CT-examination (n=379) were younger and had lower five-year mortality rate compared to those who did not have a CT-scan (n=305). Participants and non-participants in the CT-study were similar regarding tobacco smoking, diastolic and systolic blood pressure, serum cholesterol, body mass index (BMI), and frequency of stroke and myocardial infarction.

Current study includes 369 women, how responded to a question about cigarette smoking in the three midlife examinations, 1968-69, 1974-75 and 1980-81, and took part in the CT-study

For peer review only

in 2000-01 (i.e., 10 women had missing information on smoking habits). Informed consent was obtained from participants and/or their relatives.

Assessment of smoking habits

A question about tobacco smoking was asked at the examinations in 1968-69, 1974-75, and 1980-81. The response alternatives were: 0= Never smoked; 1=Smoked earlier, but not during the last 15 years; 2=Smoked earlier, but not during the last year; 3=Stopped smoking during last year; 4=Current smoking, but no deep drag; or 5=Current smoker, does deep drag. In this study, we used 3-5 to define a 'current smoker'.

CT-scans of the brain

Brain CT-scans were performed without contrast enhancement and with 10-mm continuous slices. A neurologist experienced in visual CT-rating evaluated the scans. Cortical atrophy of the temporal, frontal, parietal, and occipital lobes was categorized according to the anatomical subdivision. Severity was scored as normal, mild, and moderate-severe, according to the extent of sulcal widening.[20, 21] White matter lesions were defined as low-density areas in the periventricular subcortical white matter, and decreased density was rated as mild, moderate, and severe in relation to the attenuation of normal white matter.[22-24] The examiner was blinded to the participants' clinical characteristics.

An inter-observer agreement between the rating neurologist and a neuroradiologist was done in 130 CT-scans.[23] The intra-class correlation coefficient (ICC) was 0.49 for temporal lobe atrophy (68% concordance, p <0.001), 0.38 for frontal lobe atrophy (67% concordance, p <0.001), 0.39 for parietal lobe atrophy (68% concordance, p <0.001), 0.32 for occipital lobe atrophy (68% concordance, p <0.001), and 0.57 for presence and severity of white matter

For peer review only

lesions (55% concordance, $p<0.001$). The procedure is described in detail in former publications.[3, 23]

Assessment of other measurements

Information on alcohol consumption, body length, body height, serum cholesterol, blood pressure, antihypertensive medication, physical activity, and psychological stress, were obtained at each midlife examination. Use of alcohol consumption (beer, wine, and/or spirits) was categorized in two groups, i.e. $\leq$ once weekly and > once weekly. Body height was measured to the nearest centimeter and weight to the nearest 0.1 kg. BMI was calculated using the formula kg/m^2. Blood samples were taken after an overnight fast, and serum cholesterol concentrations were measured. Blood pressure was measured in the sitting position after five minutes' rest. Hypertension was defined as systolic blood pressure $\geq$ 150 mmHg and/or diastolic blood pressure $\geq$ 90 mmHg and/or taking antihypertensive medication. Psychological stress was defined as several periods of stress symptoms (e.g., tension, nervousness, or sleep disturbance) during last five years. Physical activity during leisure time were rated as low (< 4 hour/week) and medium/high ($\geq$ 4 hour/week). Information on stroke, myocardial infarction, heart failure and diabetes were obtained from the Swedish Hospital Discharge Register, and a diagnosis before December 31, 2002, was defined as cardio- and cerebrovascular disease (CCVD). Dementia diagnoses, according to DSM-III-R criteria, were based on information from the psychiatric examinations, close informant interviews, medical records, and the Swedish Hospital Discharge Registry, during the entire study period, from baseline 1968 to December 31, 2002.[24, 25]

Statistical methods

For peer review only

Associations between smoking and brain atrophy

Independent sample *t* tests or χ^2 tests were used to compare CT-participants and non-CT-participants. Logistic regression analyses estimated associations between midlife smoking, in 1968-69, 1974-75 and 1980-81, and cortical atrophy (no vs. mild-severe) and periventricular white matter lesions (no vs. mild-severe), in 2000-01. The associations were presented as odds ratios (OR) and 95% confidence intervals (CI). A 1st model adjusted for age and a 2nd model adjusted for age, alcohol consumption, BMI, cholesterol, hypertension, psychological stress, low physical activity, and CCVD. Persons with missing data in the BMI (n=1) and cholesterol (n=1) variables were given the mean-level of the variable, and missing data in the alcohol consumption, hypertension (n =17), and psychological stress (n=8) variables, were rated as "0" (not fulfilled criteria). All participants were therefore included in both models.

We further examined whether number of examinations when smoking was reported influenced the associations to brain atrophy and white matter lesions. The study sample was re-classified in three groups: (I) *never smokers* = never reported smoking at any examination, (II) *temporary smokers* = report of smoking at one or two examinations, and (III) *longstanding smokers* = report of smoking at all three midlife examinations. The logistic regression models were adjusted for all co-variates. The association between longstanding smoking and frontal lobe atrophy where further adjusted for white matter lesions and dementia and studied separately in the later-born cohorts (born 1922 and 1930) and the earlier-born cohorts (born 1908, 1914, and 1918).

Finally, age adjusted logistic regressions analyzed the association between alcohol consumption and longstanding smoking and frontal lobe atrophy. An interaction analysis was done to evaluate the interaction between smoking and alcohol consumption (longstanding smoking*more frequent alcohol consumption) according to frontal lobe atrophy (significance

For peer review only

level p < 0.20). The sample were stratified in lower vs. higher alcohol consumption, and logistic regressions re-analyzed the association between smoking and frontal lobe atrophy. All analyses were performed with IBM SPSS version 24 statistical software.

Patient and public involvement

Patients and/or the public were not involved in the design, or conduct, or reporting, or dissemination plans of this research

RESULTS

Characteristics of the study sample (n=369) are shown in Table 1. Mean age at baseline examination 1968-69 was 44 years (SD ± 6 years). The prevalence of cigarette smoking was 32% (n=119) in 1968-69, 29% in 1974-75 and 25% in 1980-81, baseline smoking was reported by 16% in the earlier-born cohorts (1908, 1914, and 1918) and 38% in the later-born cohorts (1922 and 1930) (Table 2). Data from the CT-study in 2000-01 (mean age 76 years SD ± 6 years) are presented in Table 3.

Cigarette smoking in 1968-69 (adjusted OR 1.85; 95% CI 1.12-3.04), in 1974-75 (OR 2.37; 95% CI 1.39-4.04) and in 1980-81 (OR 2.47; 95% CI 1.41-4.33) was associated with late-life frontal lobe atrophy (Table 4). Report of smoking at the last examination (1980-81) was also associated with parietal lobe atrophy (OR 1.99; 95% CI 1.10-3.58). There was no association between smoking and cortical atrophy in the temporal or occipital lobes, or with periventricular white matter lesions (Table 4).

Among the 351women who participated in all three midlife examinations, 65% were never-smokers, 12% reported smoking at one or two examinations, and 23% reported smoking at all

For peer review only

three examinations (Table 5). The longstanding smokers (three examinations) had higher prevalence of late-life frontal lobe atrophy (adjusted OR 2.63; 95% CI 1.44-4.78), compared to never-smokers, and the association remained after additional adjusting for periventricular white matter lesions (OR 2.57, 95% CI 1.41-4.69). There were no associations between temporary smoking (smoking in one-two examination) and frontal lobe atrophy, nor between longstanding smoking and lesions in the other cortical and white matter areas (Table 5). The adjusted ORs (95% CI) between longstanding smoking and frontal lobe atrophy were 5.31 (0.59-47.84) in the earlier-born cohorts (born 1908, 1915 and 1918) and 2.49 (1.31-4.74) in the later-born cohorts (born 1922 and 1930).

There was an interaction between smoking and alcohol consumption (longstanding smoking*higher alcohol consumption) in relation to frontal lobe atrophy (age adjusted $p_{interaction}$ 0.13). The sample were thus stratified, to explore this interaction in more details. In women with a more frequent alcohol consumption (>once weekly, n=101), longstanding smoking was associated with frontal lobe atrophy (adjusted OR 6.02, 95% CI 1.74-20.84), while no association was found in those with low alcohol consumption ($p > 0.05$). In addition, a more frequent alcohol consumption was associated with longstanding smoking (p 0.02) but not with frontal lobe atrophy (p 0.63).

Seventeen women developed dementia (Alzheimer's disease or vascular dementia) during the study period between 1968 and 2000. The association between longstanding smoking and frontal lobe atrophy remained after additional adjustment for dementia (OR 2.50, 95% CI 1.38-4.56).

DISCUSSION

For peer review only - http://bmjopen.bmj.com/site/about/guidelines.xhtml

Associations between smoking and brain atrophy

In this population-based sample of women followed over 32 years, we found that midlife tobacco smoking was associated with late-life frontal lobe atrophy. The strongest association was observed in those who reported smoking in all three midlife examinations and in women with a more frequent alcohol consumption. There was also an association between smoking in the last midlife examination (1980-81) and parietal lobe atrophy. Being a temporary smoker was not associated with late-life brain lesions. There were neither no association between smoking and atrophy in other cortical areas or in the white matter. The findings remained after controlling for several potential confounders including life-style factors, vascular diseases, and dementia.

Our findings are difficult to compare with other studies, as few earlier studies have examined the effects of smoking on structural brain lesions in longer follow-up periods. Previous studies have mostly been conducted in cross-sectional samples of young and middle-aged people or based on retrospective information on smoking habits. Among studies in younger populations, a meta-analysis of seven voxel-based morphometry (VBM) studies (n=418, median age 37) reported that chronic smokers had decreased gray matter volume in the anterior cingulate cortex,[12] i.e., the area surrounding the frontal part of corpus callosum.[27] Another meta-analysis, based on eleven VBM studies (n=1710, mean age 42), established that smokers had smaller gray matter volumes in the prefrontal cortex and larger volumes in lingual cortex, compared to non-smokers.[13] In two recent published studies it was found that current smokers had smaller frontal and temporal gray matter volume (n = 698, mean age 50),[28] and thinning in the orbitofrontal cortex, insula, and entorhinal-middle temporal regions (n=82, mean age 45).[29] Taken together, less gray matter substance, in various brain regions, has been observed in chronic smokers, and maybe most consistently in the prefrontal and frontal brain.

For peer review only

The biological mechanisms, behind frontal lobe atrophy, can have several explanations. Tobacco smoking preliminary affect the addiction-related regions in brain, [28, 30] and the frontal brain contain the highest densities of nicotinic acetylcholine receptors. [14] Nicotine interacts with the dopaminergic reward system in the forebrain,[15, 2] i.e., the mesolimbic system, [31, 32, 14] which connects the midbrain, with the basal ganglia, and the frontal cortex.[13] An acute nicotine intake causes activation of receptors, while a chronic use can lead to desensitization of receptors, due to development of tolerance.[13] Therefore, a long-term activation and exhaustion of synapses might lead to receptor damage and neurodegeneration. [14] Data from animal models shows that the neurotoxic consequences of nicotine can lead to cell loss and synaptic alterations.[33, 34. The fact that the mesolimbic pathway is part of the larger mesocortical pathway, might also explain the associations to parietal lobe atrophy,[15] which findings from this study supports.

Interestingly, the association between smoking and frontal lobe atrophy varied depending on alcohol habits, where women with a more frequent alcohol consumption had more frontal brain atrophy. Similar findings have been observed in earlier studies,[35] which suggests interaction effects between exposures linked to addiction behaviors.[15, 2] It is a well-known fact that smoking co-occurs with higher alcohol consumption,[36] and findings from studies on alcohol disorders have shown an intricate interplay between smoking use and alcohol use.[29] Alcohol has also independently been associated with several negative brain outcomes, including smaller frontal volume and desensitization in the mesolimbic system.[8, 37]

For peer review only

We found no association between smoking and white matter lesions, which was a bit unexpected. Cigarette smoking gives exposure to carbon monoxide, free radicals and free oxygen species, which have negative cerebrovascular affects, and increase the risks of arteriolosclerosis, ischemic demyelination, and lower blood flow.[28, 2, 16] The frontal cortex is vulnerable for vascular diseases, due to its reliance on multiple penetrating arteries in the underlying white matter, with few collaterals.[38, 39] When we used white matter lesions as a potential confounder between smoking and frontal lobe atrophy, the association between smoking and frontal lobe atrophy remained, i.e., it did not modify the association. One explanation could be that the study only measured periventricular lesions on CT and not the total sub-cortical volume, or more detailed blood-vessel data, as there was no such information available. The findings are however in line with several other studies that neither found associations between smoking and lower white matter volume. [28, 40, 41]

For peer review

Frontal lobe shrinkage is frequently found in cognitively healthy older people and can occur without clinical abnormalities.[23] In our sample, over half of the population had mild, moderate, or severe frontal lobe atrophy. Only a minority of these (8%) were diagnosed with dementia and no one with frontotemporal dementia. However, frontal atrophy can give rise to cognitive and psychiatric symptoms, which affecting the functioning, behavior, and well-being.[42] Only a few studies have analyzed the association between smoking and frontotemporal dementia, and none of them have found evidence of causality.[43-45]

In our study, only those who reported smoking in all three midlife examinations had higher attendance of frontal lobe atrophy, while no association was found in temporary smokers. Our findings suggest that the effect of smoking on frontal cortex decreases when smoking is stopped, as also has been shown for other adverse outcomes, such as cancer or cardiovascular

disease.[46, 47] Another observation was that the ORs were slightly higher in smokers in the 1980-81 examination, compared to the 1968-69 and 1974-75 examinations, albeit not statistically significant. This can be a sign of an increased risk of smoking at higher ages, and/or that the women who smoked in 1980 mainly were the same as the longstanding smokers (i.e., 81 of 88 persons).

The strengths of our study are the population-based sampling method, the long follow-up period with a prospective design, repeated and detailed information on smoking habits during early and late midlife, and the possibility to control for several potential confounders. However, some methodological issues need to be considered. First, we have no detailed data on smoking habits before or after the study period, and no data on number of cigarettes/per day. Second, visual rating of brain atrophy and white matter lesions on CT is a rather crude method. CT-scans are less sensitive than for example MRI to detecting brain structural changes, but similar in measuring atrophy. [48] Third, attrition is a problem in long-term prospective studies. Compared to non-participants, participants in the CT-study were younger and had a lower mortality-rate. They may thus be healthier and have less brain changes than the general population. If anything, this might likely have underestimated our findings. Fourth, we have no CT-data from the three midlife examinations (1968-80) and can therefore not exclude that some of the women had cortical atrophy already in midlife and that early subtle changes in the frontal lobes may e.g., led to addiction habits, such as smoking. Fifth, there could be a potential stigma related to the self-report of cigarettes and alcohol use. If a lower prevalence were reported, this might likely have underestimated our findings. Sixth, our study focused on smoking and other forms of nicotine use, e.g., snuff or water pipe, was not measured. However, the prevalence of these habits should have been very low among middle-aged women between 1968 and 1981. Seventh, the sample size was sometimes too

For peer review only

small for examining sub-groups, e.g., specific age-cohorts. Finally, the study includes only Caucasian women, and the results can thus not be generalised to other populations.

Conclusion

We found an association between tobacco smoking in midlife and frontal lobe atrophy in late life. The strongest association was found in women with a higher alcohol consumption. Only longstanding smoking was associated with higher risk of frontal atrophy, not sporadic smoking. Extended death of synapses and neurons in the frontal cortex might be a consequent of long-term toxic stimulation of the nicotine receptors. The findings have both clinical and public health implications, and further studies should analyse if longstanding tobacco use also as related to impaired cognitive functions.

Acknowledgements

The authors thank all members of the Prospective Population Study of Women group for their cooperation in data collection and management, and Valter Sundh for statistical assistance.

Contributorship statement

All authors (L.J., X.G., S.S., M.M.F., S.K., A.Z. and, I.S.) contributed to the conception or design of the work and drafted and approved the final version. All agreed to be accountable for all aspects of the work in ensuring that questions related to the accuracy or integrity of any part of the work are appropriately investigated and resolved.

Competing interests

None declared.

For peer review only

Funding

The study was financed by grants from the Swedish state under the agreement between the Swedish government and the county councils, the ALF-agreement (ALF 716681), Swedish Research Council for Health, Working Life and Wellfare (2004-0145, 2006-0596, 2008-1111, 2010-0870, 2012-1138, 2013-1202, 2018-00471, AGECAP 2013-2300), Hjärnfonden (FO2014-0207, FO2016-0214, FO2018-0214, FO2019-0163), The Alzheimer's Association Stephanie B. Overstreet Scholars (IIRG-00-2159), The Alzheimer's Association Zenith Award (ZEN-01-3151), and Swedish Research Council (2005-8460, 2007-7462, 2012-5041, 2015-02830, 2019-01096, 2013-8717, NEAR 2017-00639).

Data sharing statement

Data are available upon reasonable request.

Ethics approval statement

The study was approved by the Ethics Committee for Medical Research at the University of Gothenburg.

For peer review only

REFERENCES

1. Ekpu VU, Brown AK. The Economic Impact of Smoking and of Reducing Smoking Prevalence: Review of Evidence. *Tob Use Insights* 2015;8,1-35.doi:10.4137/TUI.S15628

2. Swan GE, Lessov-Schlaggar CN The effects of tobacco smoke and nicotine on cognition and the brain. *Neuropsychol Rev* 2007;17(3),259-273.doi:10.1007/s11065-007-9035-9

3. Johansson L, Skoog I, Gustafson DR et al..Midlife psychological distress associated with late-life brain atrophy and white matter lesions: a 32-year population study of women. *Psychosom Med* 2012;74(2),120-125.doi:PSY.0b013e318246eb10

4. Pini L, Pievani M, Bocchetta M et al.. Brain atrophy in Alzheimer's Disease and aging. *Ageing Res Rev* 2016;30,25-48.doi:10.1016/j.arr.2016.01.002

5. Pantoni L. Cerebral small vessel disease: from pathogenesis and clinical characteristics to therapeutic challenges. *Lancet Neurol* 2010;9(7),689-701.doi:10.1016/S1474-4422(10)70104-6

6. Agosta F, Vossel KA, Miller BL, et al.. Apolipoprotein E epsilon4 is associated with disease-specific effects on brain atrophy in Alzheimer's disease and frontotemporal dementia. *Proc Natl Acad Sci U S A* 2009;106(6), 2018-2022.doi:10.1073/pnas.0812697106

7. Gazdzinski S, Kornak J, Weiner MW et al.. Body mass index and magnetic resonance markers of brain integrity in adults. *Ann Neurol* 2008;63(5),652-657.doi:10.1002/ana.21377

For peer review only

8. Kubota M, Nakazaki S, Hirai S et al.. Alcohol consumption and frontal lobe shrinkage: study of 1432 non-alcoholic subjects. *J Neurol Neurosurg Psychiatry*2001;71(1), 104-106. Retrieved from https://www.ncbi.nlm.nih.gov/pubmed/11413273

9. Lou B, Chen M, Luo X et al.. Reduced right frontal fractional anisotropy correlated with early elevated plasma LDL levels in obese young adults. *PLoS One* 2014;9(10),e108180.doi:10.1371/journal.pone.0108180

10. Zhang Y, Zhang X, Zhang J et al.. Gray matter volume abnormalities in type 2 diabetes mellitus with and without mild cognitive impairment. *Neurosci Lett* 2014;562,1-6.doi:10.1016/j.neulet.2014.01.006

11. Gray JC, Thompson M, Bachman C et al.. Associations of cigarette smoking with gray and white matter in the UK Biobank. *Neuropsychopharmacology* 2020;45(7):1215-1222. doi: 10.1038/s41386-020-0630-2

12. Pan P, Shi H, Zhong J et al.. Chronic smoking and brain gray matter changes: evidence from meta-analysis of voxel-based morphometry studies. *Neurol Sci* 2013;34(6),813-817.doi:10.1007/s10072-012-1256-x

13. Zhong J, Shi H, Shen Y et al.. Voxelwise meta-analysis of gray matter anomalies in chronic cigarette smokers. *Behav Brain Res* 2016;311,39-45.doi:10.1016/j.bbr.2016.05.016

For peer review only

14. Kober H, Deleone CM. Smoking and Neuroimaging: A Review. *Curr Cardiovasc Risk Rep* 2011;5(6),484-491.doi:10.1007/s12170-011-0201-5

15. Jasinska AJ, Zorick T, Brody AL et al.. Dual role of nicotine in addiction and cognition: a review of neuroimaging studies in humans. *Neuropharmacology* 2014;84,111-122.doi:10.1016/j.neuropharm.2013.02.015

16. Tomonaga M, Yamanouchi H, Tohgi H et al.. Clinicopathologic study of progressive subcortical vascular encephalopathy (Binswanger type) in the elderly. *J Am Geriatr Soc* 1982;30(8),524-529.

17. Durazzo TC, Meyerhoff DJ. Neurobiological and neurocognitive effects of chronic cigarette smoking and alcoholism. *Front Biosci* 2007;12,4079-4100.doi:10.2741/2373

18. Bengtsson C, Blohme G, Hallberg L, et al.. The study of women in Gothenburg 1968-1969--a population study. General design, purpose and sampling results. *Acta Med Scand* 1973;193(4), 311-318.

19. Guo X, Skoog I, Matousek M et al.. A population-based study on motor performance and white matter lesions in older women. *J Am Geriatr Soc* 2000;48(8),967-970.

20. De Leon MJ, Ferris SH, George AE, et al.. Computed tomography evaluations of brain-behavior relationships in senile dementia of the Alzheimer's type. *Neurobiology of Aging* 1980;1(1),69-79.

For peer review only

21. Thompson PM, Hayashi KM, de Zubicaray G et al.. Dynamics of gray matter loss in Alzheimer's disease. *J Neurosci* 2003;33(3),994-1005.doi:23/3/994 [pii]

22. Gustafson D, Lissner L, Bengtsson C et al.. A 24-year follow-up of body mass index and cerebral atrophy. *Neurology* 2004;63(10),1876-1881.doi:63/10/1876[pii]

23. Simoni M, Pantoni L, Pracucci G et al.. Prevalence of CT-detected cerebral abnormalities in an elderly Swedish population sample. *Acta Neurol Scand* 2008;118(4),260-267. doi:ANE1010

24. Skoog I, Palmertz B, Andreasson LA The prevalence of white-matter lesions on computed tomography of the brain in demented and nondemented 85-year-olds. *J Geriatr Psychiatry Neurol* 1994;7(3),169-175

25. Guo X, Waern M, Sjogren K et al.. Midlife respiratory function and Incidence of Alzheimer's disease: a 29-year longitudinal study in women. *Neurobiology of Aging* 2007;28(3),343-350.doi:S0197-4580(06)00041-8

26. Skoog I, Nilsson L, Palmertz B et al.. A population-based study of dementia in 85-year-olds. *N Engl J Med* 1993;328(3),153-158.doi:10.1056/NEJM199301213280301

27. Taylor SF, Martis B, Fitzgerald KD et al.. Medial frontal cortex activity and loss-related responses to errors. *J Neurosci* 2006;26(15),4063-4070.doi:10.1523/JNEUROSCI.4709-05.2006

For peer review only

28. Elbejjani M., Auer R, Jacobs DR et al..Cigarette smoking and gray matter brain volumes in middle age adults: the CARDIA Brain MRI sub-study. *Transl Psychiatry* 2019;9(1),78.doi:10.1038/s41398-019-0401-1

29. Durazzo TC, Meyerhoff DJ, Yoder KK. Cigarette smoking is associated with cortical thinning in anterior frontal regions, insula and regions showing atrophy in early Alzheimer's Disease. *Drug Alcohol Depend* 2018;192, 277-284.doi:10.1016/j.drugalcdep.2018.08.009

30. Wang K, Yang J, Zhang S et al.. The neural mechanisms underlying the acute effect of cigarette smoking on chronic smokers. *PLoS One* 2014;9(7),e102828.doi:10.1371/journal.pone.0102828

31. Dinur-Klein L, Dannon P, Hadar A, et al.. Smoking cessation induced by deep repetitive transcranial magnetic stimulation of the prefrontal and insular cortices: a prospective, randomized controlled trial. *Biol Psychiatry* 2014;76(9),742-749.doi:10.1016/j.biopsych.2014.05.020

32. Hartwell KJ, Prisciandaro JJ, Borckardt J et al.. Real-time fMRI in the treatment of nicotine dependence: a conceptual review and pilot studies. *Psychol Addict Behav* 2013;27(2),501-509.doi:10.1037/a0028215

33. Chen WJ, Edwards RB, Romero RD, et al.. Long-term nicotine exposure reduces Purkinje cell number in the adult rat cerebellar vermis. *Neurotoxicol Teratol* 2003; 25(3), 329-334. doi:10.1016/s0892-0362(02)00350-1

For peer review only

34. Trauth JA, McCook EC, Seidler FJ Modeling adolescent nicotine exposure: effects on cholinergic systems in rat brain regions. *Brain Res* 2000;873(1),18-25.doi:10.1016/s0006-8993(00)02465-3

35. Elbejjani M, Auer R, Dolui, S et al.. Cigarette smoking and cerebral blood flow in a cohort of middle-aged adults. *J Cereb Blood Flow Metab* 2019;39(7), 1247-1257.doi:10.1177/0271678X18754973

36. Room R Smoking and drinking as complementary behaviours. *Biomed Pharmacother* 2004;58(2),111-115.doi:10.1016/j.biopha.2003.12.003

37. Rando K, Hong KI, Bhagwagar Z et al.. Association of frontal and posterior cortical gray matter volume with time to alcohol relapse: a prospective study. *Am J Psychiatry* 2011;168(2),183-192.doi:10.1176/appi.ajp.2010.10020233

38. Chen Y, Chen X, Xiao W, et al.. Frontal lobe atrophy is associated with small vessel disease in ischemic stroke patients. *Clin Neurol Neurosurg* 2009;111(10), 852-857.doi:10.1016/j.clineuro.2009.08.019

39. Foster V, Oakley AE, Slade JY et al.. Pyramidal neurons of the prefrontal cortex in post-stroke, vascular and other ageing-related dementias. *Brain* 2014;137(Pt 9),2509-2521.doi:10.1093/brain/awu172

For peer review only

40. Fritz HC, Wittfeld K, Schmidt CO et al.. Current smoking and reduced gray matter volume-a voxel-based morphometry study. *Neuropsychopharmacology* 2014;39(11),2594-2600.doi:10.1038/npp.2014.112

41. Gallinat J, Meisenzahl E, Jacobsen LK et al.. Smoking and structural brain deficits: a volumetric MR investigation. *Eur J Neurosci* 2006;24(6),1744-1750.doi:10.1111/j.1460-9568.2006.05050.x

42. Chan DK, Reutens S, Liu DK, et al.. Frontotemporal dementia - features, diagnosis and management. *Aust Fam Physician* 2011;40(12),968-972

43. Kalkonde YV, Jawaid A, Qureshi S et al.. Medical and environmental risk factors associated with frontotemporal dementia: a case-control study in a veteran population. *Alzheimers Dement* 2012;8(3),204-210.doi:10.1016/j.jalz.2011.03.011

44. Rasmussen Eid H, Rosness TA, Bosnes O et al.. Smoking and Obesity as Risk Factors in Frontotemporal Dementia and Alzheimer's Disease: The HUNT Study. *Dement Geriatr Cogn Dis Extra* 2019;9(1),1-10.doi:10.1159/000495607

45. Tremolizzo L, Bianchi E, Susani E et al.. Voluptuary Habits and Risk of Frontotemporal Dementia: A Case Control Retrospective Study. *J Alzheimers Dis* 2017;60(2),335-340.doi:10.3233/JAD-170260

For peer review only

46. Lee H, Son YJ. Influence of Smoking Status on Risk of Incident Heart Failure: A Systematic Review and Meta-Analysis of Prospective Cohort Studies. *Int J Environ Res Public Health* 2009;16(15).doi:10.3390/ijerph16152697

47. Parsons A, Daley A, Begh R et al.. Influence of smoking cessation after diagnosis of early stage lung cancer on prognosis: systematic review of observational studies with meta-analysis. *BMJ* 2010;340,b5569.doi:10.1136/bmj.b5569

48. Frisoni GB. Structural imaging in the clinical diagnosis of Alzheimer's disease: problems and tools. *J Neurol Neurosurg Psychiatry* 2001;70(6),711-718.

For peer review only

Table 1 Characteristics of the study sample (n=369)

Baseline age (years), mean ± SD	43.8 ± 5.6
Birth year 1908, n (%)	2 (0.5)
Birth year 1914, n (%)	19 (5.1)
Birth year 1918, n (%)	80 (21.7)
Birth year 1922, n (%)	105 (28.5)
Birth year 1930, n (%)	163 (44.2)
Current smoking, n (%)	
Examination 1968-68	119 (32.2)
Examination 1974-75	104 (28.8)
Examination 1980-81	88 (25.0)
Co-variates	
Alcohol consumption (> once weekly) [a], n (%)	101 (27.4)
Body mass index (BMI) [ad], mean ± SD	23.46 ± 0.17
Cardio- and cerebrovascular diseases (CCVD)[e], n (%)	53 (14.4)
Cholesterol (mmol/l) [ad], mean ± SD	6.59 ± 0.08
Hypertension [ac], n (%)	77 (20.9)
Psychological stress [ab], n (%)	67 (18.2)
Low physical activity (< 4 hour/week) [a], n (%)	51 (13.8)
Dementia[e], n (%)	17 (4.6)

[a] Measured at baseline, 1968

[b] 8 with missing information

[c] 17 with missing information

[d] 1 with missing information

[e] Measured until December 31, 2002

For peer review only

Table 2 Prevalence of cigarette smoking in midlife examinations, presented by birth cohort

	Born 1908	Born 1914	Born 1918	Born 1922	Born 1930
Current smoking 1968-69, n (%)	0 (0)	2 (10.5)	16 (20.0)	38 (36.2)	63 (38.7)
Currant smoking 1974-75, n (%)	0 (0)	2 (10.5)	14 (18.2)	28 (26.9)	60 (37.7)
Current smoking 1980-81, n (%)	0 (0)	2 (10.5)	11 (14.9)	23 (22.8)	52 (33.3)

For peer review only

Table 3 Prevalence of cortical atrophy and white matter lesions on CT-scans in 2000-01, presented by birth cohort

	Born 1908 (n=2)	Born 1914 (n=19)	Born 1918 (n=80)	Born 1922 (n=105)	Born 1930 (n=163)	Total sample (n=369)
Temporal lobe atrophy, n (%)	2 (100)	17 (89.5)	57 (71.3)	57 (54.3)	52 (31.9)	185 (50.1)
Frontal lobe atrophy, n (%)	1 (50)	17 (89.5)	57 (71.3)	62 (59.0)	60 (36.8)	197 (53.4)
Parietal lobe atrophy, n (%)	1 (50)	15 (78.9)	42 (52.5)	49 (46.7)	27 (16.6)	134 (36.3)
Occipital lobe atrophy, n (%)	1 (50)	12 (63.2)	33 (41.3)	27 (25.7)	10 (6.1)	83 (22.5)
White matter lesions, n (%)	2 (100)	15 (78.9)	56 (70.0)	73 (69.5)	66 (40.5)	212 (57.5)

CT: Computerized tomography

For peer review only

Table 4 Associations between midlife smoking and late-life structural brain changes on CT-scans in 2000

	Tobacco smoking in 1968 (n=119)	Tobacco smoking in 1974 (n=104)	Tobacco smoking in 1980 (n=88)
Temporal lobe atrophy			
No. of cases	53	50	45
OR_1 (95% CI)	0.93 (0.58-1.49)	1.23 (0.75-2.03)	1.53 (0.90-2.61)
OR_2 (95% CI)	0.95 (0.58-1.56)	1.27 (0.75-2.13)	1.60 (0.92-2.79)
Frontal lobe atrophy			
No. of cases	69	63	54
OR_1 (95% CI)	**1.80 (1.11-2.92)**	**2.24 (1.34-3.73)**	**2.30 (1.34-3.95)**
OR_2 (95% CI)	**1.85 (1.12-3.04)**	**2.37 (1.39-4.04)**	**2.47 (1.41-4.33)**
Parietal lobe atrophy			
No. of cases	42	36	34
OR_1 (95% CI)	1.32 (0.80-2.19)	1.37 (0.81-2.34)	**1.89 (1.07-3.33)**
OR_2 (95% CI)	1.38 (0.81-2.34)	1.37 (0.79-2.39)	**1.99 (1.10-3.58)**
Occipital lobe atrophy			
No. of cases	24	21	20
OR_1 (95% CI)	1.21 (0.67-2.20)	1.35 (0.73-2.55)	1.75 (0.90-3.39)
OR_2 (95% CI)	1.25 (0.67-2.32)	1.35 (0.70-2.61)	1.84 (0.92-3.67)
White matter lesions			
No. of cases	68	61	52
OR_1 (95% CI)	1.26 (0.79-2.02)	1.41 (0.86-2.32)	1.51 (0.89-2.56)
OR_2 (95% CI)	1.43 (0.74-2.75)	1.83 (0.94-3.57)	1.27 (0.62-2.64)

OR (95%): odds ratio (95% confidence interval)

CT: Computerized tomography

OR_1 adjusted for age

OR_2 adjusted for age, alcohol consumption, BMI, CCVD, cholesterol, hypertension, physical activity, and psychological stress

For peer review only

Table 5 Associations between midlife smoking in multiple examinations, between 1968-80, and late-life structural brain changes on CT-scans in 2000 (n=351)

	No tobacco smoking in any examination (n=227)	Tobacco smoking in 1-2 examination (n=43)	Tobacco smoking in 3 examinations (n=81)
Temporal lobe atrophy<br>No. of cases	121	17	40
OR_1 (95% CI)	1.0 (ref.)	0.74 (0.37-1.51)	1.24 (0.71-2.17)
OR_2 (95% CI)	1.0 (ref.)	0.76 (0.37-1.56)	1.28 (0.72-2.30)
Frontal lobe atrophy<br>No. of cases	118	19	51
OR_1 (95% CI)	1.0 (ref.)	0.96 (0.48-1.91)	**2.36 (1.36-4.18)**
OR_2 (95% CI)	1.0 (ref.)	1.00 (0.49-2.04)	**2.63 (1.44-4.78)**
Parietal lobe atrophy<br>No. of cases	84	12	31
OR_1 (95% CI)	1.0 (ref.)	0.94 (0.44-2.01)	1.69 (0.94-3.03)
OR_2 (95% CI)	1.0 (ref.)	0.99 (0.45-2.18)	1.82 (0.98-3.39)
Occipital lobe atrophy<br>No. of cases	55	7	18
OR_1 (95% CI)	1.0 (ref.)	0.99 (0.39-2.50)	1.57 (0.79-3.12)
OR_2 (95% CI)	1.0 (ref.)	0.96 (0.37-2.46)	1.60 (0.77-3.32)
White matter lesions<br>No. of cases	131	22	49
OR_1 (95% CI)	1.0 (ref.)	0.97 (0.49-1.92)	1.55 (0.89-2.69)
OR_2 (95% CI)	1.0 (ref.)	1.44 (0.56-3.72)	1.53 (0.71-3.29)

OR (95%): odds ratio (95% confidence interval)

CT: Computerized tomography

OR_1 adjusted for age

OR_2 adjusted for age, alcohol consumption, BMI, CCVD, cholesterol, hypertension, physical activity, and psychological stress

For peer review only

STROBE Statement—Checklist of items that should be included in reports of ***cohort studies***

	Item No	Recommendation	Page No
**Title and abstract**	1	(*a*) Indicate the study's design with a commonly used term in the title or the abstract<br>(*b*) Provide in the abstract an informative and balanced summary of what was done and what was found	1
**Introduction**			
Background/rationale	2	Explain the scientific background and rationale for the investigation being reported	2,3,4
Objectives	3	State specific objectives, including any prespecified hypotheses	2,3,4
**Methods**			
Study design	4	Present key elements of study design early in the paper	4,5,6
Setting	5	Describe the setting, locations, and relevant dates, including periods of recruitment, exposure, follow-up, and data collection	4,5,6
Participants	6	(*a*) Give the eligibility criteria, and the sources and methods of selection of participants. Describe methods of follow-up<br>(*b*) For matched studies, give matching criteria and number of exposed and unexposed	4,5,6
Variables	7	Clearly define all outcomes, exposures, predictors, potential confounders, and effect modifiers. Give diagnostic criteria, if applicable	4-7
Data sources/ measurement	8*	For each variable of interest, give sources of data and details of methods of assessment (measurement). Describe comparability of assessment methods if there is more than one group	4-7
Bias	9	Describe any efforts to address potential sources of bias	4-7, 11
Study size	10	Explain how the study size was arrived at	4-5
Quantitative variables	11	Explain how quantitative variables were handled in the analyses. If applicable, describe which groupings were chosen and why	4-7
Statistical methods	12	(*a*) Describe all statistical methods, including those used to control for confounding<br>(*b*) Describe any methods used to examine subgroups and interactions<br>(*c*) Explain how missing data were addressed<br>(*d*) If applicable, explain how loss to follow-up was addressed<br>(*e*) Describe any sensitivity analyses	7
**Results**			
Participants	13*	(a) Report numbers of individuals at each stage of study—eg numbers potentially eligible, examined for eligibility, confirmed eligible, included in the study, completing follow-up, and analysed<br>(b) Give reasons for non-participation at each stage<br>(c) Consider use of a flow diagram	7,8
Descriptive data	14*	(a) Give characteristics of study participants (eg demographic, clinical, social) and information on exposures and potential confounders<br>(b) Indicate number of participants with missing data for each variable of interest<br>(c) Summarise follow-up time (eg, average and total amount)	7,8
Outcome data	15*	Report numbers of outcome events or summary measures over time	7,8

For peer review only

Main results	16	(*a*) Give unadjusted estimates and, if applicable, confounder-adjusted estimates and their precision (eg, 95% confidence interval). Make clear which confounders were adjusted for and why they were included<br>(*b*) Report category boundaries when continuous variables were categorized<br>(*c*) If relevant, consider translating estimates of relative risk into absolute risk for a meaningful time period	7-9
Other analyses	17	Report other analyses done—eg analyses of subgroups and interactions, and sensitivity analyses	7,8
**Discussion**			
Key results	18	Summarise key results with reference to study objectives	8-12
Limitations	19	Discuss limitations of the study, taking into account sources of potential bias or imprecision. Discuss both direction and magnitude of any potential bias	12
Interpretation	20	Give a cautious overall interpretation of results considering objectives, limitations, multiplicity of analyses, results from similar studies, and other relevant evidence	12
Generalisability	21	Discuss the generalisability (external validity) of the study results	12
**Other information**			
Funding	22	Give the source of funding and the role of the funders for the present study and, if applicable, for the original study on which the present article is based	12

*Give information separately for exposed and unexposed groups.

Note: An Explanation and Elaboration article discusses each checklist item and gives methodological background and published examples of transparent reporting. The STROBE checklist is best used in conjunction with this article (freely available on the Web sites of PLoS Medicine at http://www.plosmedicine.org/, Annals of Internal Medicine at http://www.annals.org/, and Epidemiology at http://www.epidem.com/). Information on the STROBE Initiative is available at http://www.strobe-statement.org.

For peer review only

BMJ Open

Longstanding smoking associated with frontal brain lobe atrophy: A 32-year follow-up study in women

Journal:	*BMJ Open*
Manuscript ID	bmjopen-2023-072803.R1
Article Type:	Original research
Date Submitted by the Author:	07-Jul-2023
Complete List of Authors:	Johansson, Lena; University of Gothenburg Institute of Neuroscience and Physiology, Department of Psychiatry and Neurochemistry, Centre for Ageing and Health (AgeCap); Sahlgrenska universitetssjukhuset, Department of Addiction and Dependency, Sahlgrenska University Hospital, Gothenburg, Sweden<br>Guo, Xinxin; University of Gothenburg Institute of Neuroscience and Physiology, Department of Psychiatry and Neurochemistry, Sahlgrenska Academy, Centre for Ageing and Health (AgeCap)<br>Sacuiu, S; University of Gothenburg Institute of Neuroscience and Physiology, Department of Psychiatry and Neurochemistry, Sahlgrenska Academy, Centre for Ageing and Health (AgeCap)<br>Fässberg, Madeleine; University of Gothenburg Institute of Neuroscience and Physiology, Department of Psychiatry and Neurochemistry, Sahlgrenska Academy, Centre for Ageing and Health (AgeCap)<br>Kern, Silke; University of Gothenburg Institute of Neuroscience and Physiology, Department of Psychiatry and Neurochemistry, Sahlgrenska Academy, Centre for Ageing and Health (AgeCap)<br>Zettergren, Anna; University of Gothenburg Institute of Neuroscience and Physiology, Department of Psychiatry and Neurochemistry, Sahlgrenska Academy, Centre for Ageing and Health (AgeCap)<br>Skoog, Ingmar; University of Gothenburg Institute of Neuroscience and Physiology, Department of Psychiatry and Neurochemistry, Sahlgrenska Academy, Centre for Ageing and Health (AgeCap)
**Primary Subject Heading**:	Smoking and tobacco
Secondary Subject Heading:	Neurology, Pathology, Epidemiology
Keywords:	EPIDEMIOLOGIC STUDIES, NEUROPATHOLOGY, Old age psychiatry < PSYCHIATRY

SCHOLARONE™
Manuscripts

I, the Submitting Author has the right to grant and does grant on behalf of all authors of the Work (as defined in the below author licence), an exclusive licence and/or a non-exclusive licence for contributions from authors who are: i) UK Crown employees; ii) where BMJ has agreed a CC-BY licence shall apply, and/or iii) in accordance with the terms applicable for US Federal Government officers or employees acting as part of their official duties; on a worldwide, perpetual, irrevocable, royalty-free basis to BMJ Publishing Group Ltd ("BMJ") its licensees and where the relevant Journal is co-owned by BMJ to the co-owners of the Journal, to publish the Work in this journal and any other BMJ products and to exploit all rights, as set out in our licence.

The Submitting Author accepts and understands that any supply made under these terms is made by BMJ to the Submitting Author unless you are acting as an employee on behalf of your employer or a postgraduate student of an affiliated institution which is paying any applicable article publishing charge ("APC") for Open Access articles. Where the Submitting Author wishes to make the Work available on an Open Access basis (and intends to pay the relevant APC), the terms of reuse of such Open Access shall be governed by a Creative Commons licence – details of these licences and which Creative Commons licence will apply to this Work are set out in our licence referred to above.

Other than as permitted in any relevant BMJ Author's Self Archiving Policies, I confirm this Work has not been accepted for publication elsewhere, is not being considered for publication elsewhere and does not duplicate material already published. I confirm all authors consent to publication of this Work and authorise the granting of this licence.

For peer review only

Longstanding smoking associated with frontal brain lobe atrophy: A 32-year follow-up study in women

Lena Johansson [ab] Xinxin Guo [a], Simona Sacuiu [a], Madeleine Mellqvist Fässberg [a], Silke Kern [a], Anna Zettergren [a], Ingmar Skoog [a]

Affiliations

[a] Department of Psychiatry and Neurochemistry, Sahlgrenska Academy, Centre for Ageing and Health (AgeCap) at the University of Gothenburg, Sweden

[b] Department of Addiction and Dependency, Sahlgrenska University Hospital, Gothenburg, Sweden

Address for correspondence: Lena Johansson, University of Gothenburg and Sahlgrenska University Hospital, Institute of Neuroscience and Physiology, Department of Psychiatry and Neurochemistry, Unit of Psychiatric Epidemiology, Wallinsgatan 6, 431 41 Mölndal, Sweden. Tel: +46 31 343 86 47 Fax: +46 31 776 04 03 E-mail: lena.johansson@neuro.gu.se

Abbreviations: BMI: body mass index, CCVD: cardio- and cerebrovascular diseases, CI: confidence intervals, CT: computerized tomography, MRI: magnetic resonance imaging, OR: odds ratios, VBM: voxel-based morphometry

For peer review only

ABSTRACT

Objective To examine the association between midlife tobacco smoking and late-life brain atrophy and white matter lesions.

Methods The study includes 369 women from the Prospective Population Study of Women in Gothenburg, Sweden. Cigarette smoking was reported at baseline 1968 (mean age = 44) and at follow-up in 1974-75 and 1980-81. Brain computerized tomography (CT) was conducted 32 years after baseline examination (mean age = 76), to evaluate cortical atrophy and white matter lesions. Multiple logistic regressions estimated associations between midlife smoking and late-life brain lesions. The final analyses were adjusted for alcohol consumption and several other covariates.

Results Smoking in 1968-69 (adjusted OR 1.85; 95% CI 1.12-3.04), in 1974-75 (OR 2.37; 95% CI 1.39-4.04), and in 1980-81 (OR 2.47; 95% CI 1.41-4.33) were associated with late-life frontal lobe atrophy (2000-01). The strongest association was observed in women who reported smoking at all three midlife examinations (OR 2.63; 95% CI 1.44-4.78) and in those with more frequent alcohol consumption (OR 6.02; 95% CI 1.74-20.84). Smoking in 1980-81 was also associated with late-life parietal lobe atrophy (OR 1.99 95%; CI 1.10-3.58). There were no associations between smoking and atrophy in the temporal or occipital lobe, or with white matter lesions.

Conclusion Longstanding tobacco smoking was mainly associated with atrophy in frontal lobe cortex. A long-term stimulation of nicotine receptors in the frontal neural pathway might be harmful for targeted brain cell.

For peer review only

STRENGTHS AND LIMITATIONS OF THIS STUDY

⇒ Analyzes were done in a prospective population-based dataset with repeated information on smoking habits and other variables during participants middle-age.

⇒ There were no available data on number of cigarettes/per day, smoking habits before and the study period, or other forms of nicotine use.

⇒ It can be a potential stigma related to the self-reporting of cigarettes and alcohol use.

⇒ Visual rating of brain atrophy and white matter lesions on CT is a rather crude method, compared to e.g., magnetic resonance tomography (MRI).

⇒ CT-data from earlier in life were not available. Cortical atrophy in midlife could have led to addiction habits such as smoking, i.e., reverse causality.

For peer review only

INTRODUCTION

Cigarette smoking continues to be one of the most common addictive habits globally and is the single greatest cause of preventable death.[1] Tobacco consists of thousands of harmful compounds, of which many are directly toxic to the brain and the central nervous system (CNS).[2] The short-term effect on the brain is rather well known, but less research has been done regarding the long-term consequences.

Brain lesions increase with age, often without an identified aetiology.[3, 4] Cortical atrophy is due to advanced loss of neurons in gray matter areas, expressed either generally in the entire cortex, or partial, in specific brain areas. The neurodegeneration can be a result of proteopathies, inflammatory processes, or oxidative stress.[4] Sub-cortical lesions are most often related to cerebrovascular damages, such as small-vessel disease or brain infarcts, and relate to a widespread damage on the cell axons in white matter areas.[5] The consequences of extended cortical and sub-cortical shrinkage can be expressed as cognitive and functional impairment.[4]

A number of genetic and life-style factors have been associated with structural brain changes.[6-10] Tobacco use poses a risk to the brain due to the exposure of toxic chemicals, but few studies have examined long-term associations between cigarette smoking and late-life brain lesions.[11, 12, 13] The most addictive and intoxicating ingredient in tobacco is nicotine, an alkaloid that mainly binds to acetylcholine receptors in CNS,[14] and interacts with the dopaminergic reward system.[15, 2] Chronic tobacco smoking increases the amount of nicotine receptors in the brain and changes the structure and function of involved neurons.[2] Nicotine has also negative vascular effects to the brain, due to arteriolosclerosis and increased blood pressure.[2, 16]

For peer review only

In this study, we analysed the relation between midlife tobacco smoking and the presence of gray matter atrophy and white matter lesions on computerized tomography (CT), in late-life, in a population-based sample of 369 women followed over 32 years. We had an extra focus on alcohol consumption, given its high co-morbidity with smoking and emerging findings regarding their interactions.[17] The study adjusts for several important covariates, e.g., vascular risk factors.

METHODS

Study population

The study is part of the Prospective Population Study of Women in Gothenburg which was initiated in 1968 with a comprehensive examination of 1462 women (participation rate 90%) born in 1908, 1914, 1918, 1922 and 1930.[18, 19] Individuals were systematically sampled from the Swedish Population Register, based on specific birth dates, to yield a representative sample. Follow-ups were performed in 1974-75, 1980-81, 1992-93, and 2000-01, with participation rates among survivors of 91%, 83%, 70%, and 71%, respectively. In 2000-01 all participants (n=684) were invited for brain CT-scan examination. Participants and non-participants in the CT-study (n=379 vs. n=305, participation rate 55%) were similar regarding tobacco smoking, diastolic and systolic blood pressure, serum cholesterol, body mass index (BMI), and frequency of stroke and myocardial infarction. However, CT-participants were younger and had lower five-year mortality rate compared to those who declined to take part.

The current study includes 369 women, who responded to the question about cigarette smoking in any of the three midlife examinations, 1968-69, 1974-75 and 1980-81, and took

For peer review only

part in the CT-study in 2000-01 (10 women were excluded due to missing information on smoking habits). Informed consent was obtained from participants and/or their relatives.

Assessment of smoking habits

A question about tobacco smoking habits was asked and the alternative answers were: 0= Never smoked; 1=Smoked earlier, but not during the last 15 years; 2=Stopped smoking 1-15 years ago; 3=Stopped smoking 0-12 month ago; 4=Current smoking, but no deep drag; or 5=Current smoker, does deep drag. In this study, we used 3-5 to define a 'current smoker'.

Assessment of brain CT-scans

Brain CT-scans were performed without contrast enhancement and with 10-mm contiguous slices. A neurologist experienced in visual CT-rating evaluated all images. [20] Cortical atrophy of the temporal, frontal, parietal, and occipital lobes was categorized according to the anatomical subdivision. Severity was scored as normal, mild, and moderate-severe, according to the extent of sulcal widening.[20, 21] White matter lesions were defined as low-density areas in the periventricular subcortical white matter, and decreased density was rated as mild, moderate, and severe in relation to the attenuation of normal white matter.[20-24] The examiner was blinded to the participants' clinical characteristics.

An inter-observer agreement between the rating neurologist and a neuroradiologist was done in 130 scans.[20] The interrater Kappa value (κ) was 0.61 for temporal lobe atrophy (78% concordance), 0.53 for frontal lobe atrophy (74% concordance), 0.50 for parietal lobe atrophy (80% concordance), 0.62 for occipital lobe atrophy (90% concordance), and 0.65 for presence and severity of white matter lesions (82% concordance), i.e., an interrater reliability between moderate (κ = 0.50) and substantial (κ = 0.65).[20]

For peer review only

Assessment of other measurements

Information on alcohol consumption, body length, body height, serum cholesterol, blood pressure, antihypertensive medication, physical activity, and psychological stress, were obtained at each midlife examination. Use of alcohol consumption (beer, wine, and/or spirits) was categorized in two groups, i.e., ≤ once weekly and > once weekly. Body height was measured to the nearest centimeter and weight to the nearest 0.1 kg. BMI was calculated using the formula kg/m^2. Blood samples were taken after an overnight fast, and serum cholesterol concentrations were measured. Blood pressure was measured in the sitting position after five minutes' rest. Hypertension was defined as systolic blood pressure ≥ 150 mmHg and/or diastolic blood pressure ≥ 90 mmHg and/or taking antihypertensive medication. Psychological stress was defined as several periods of stress symptoms (e.g., tension, nervousness, or sleep disturbance) during last five years. Physical activity during leisure time was rated as low (< 4 hour/week) and medium/high (≥ 4 hour/week). Information on stroke, myocardial infarction, heart failure and diabetes was obtained from the Swedish Hospital Discharge Register, and any of these diagnosis before December 31, 2002, were defined as cardio- and cerebrovascular disease (CCVD). Dementia was diagnosed according to the DSM-III-R criteria, based on information from the psychiatric examinations, close informant interviews, medical records, and the Swedish Hospital Discharge Registry, during the entire study period, i.e., from baseline in 1968 to December 31, 2002.[24, 25, 26]

Statistical methods

Independent sample *t* tests or χ^2 tests were used to compare CT-participants and non-CT-participants. Logistic regression analyses estimated associations between report of midlife smoking, in 1968-81, and cortical atrophy (no vs. mild-severe) and periventricular white

For peer review only

matter lesions (no vs. mild-severe) at CT-examination in 2000-01. The associations are presented as odds ratios (OR) and 95% confidence intervals (CI), adjusted for age in 1st model. In a 2nd model we adjusted for age, alcohol consumption, BMI, cholesterol, hypertension, psychological stress, low physical activity, and CCVD. Persons with missing data in the BMI (n=1) and cholesterol (n=1) variables were given the mean-level of the variable, and missing data in the alcohol consumption, hypertension (n =17), and psychological stress (n=8) variables, were rated as "0" (not fulfilled criteria). Hence, all participants were included in both models.

We further examined whether the number of examinations when smoking was reported influenced the associations to brain atrophy and white matter lesions. The study sample was re-classified into three groups: (I) *never smokers* = never reported smoking at any examination, (II) *temporary smokers* = report of smoking at one or two examinations, and (III) *longstanding smokers* = report of smoking at all three midlife examinations. The association between longstanding smoking and frontal lobe atrophy were analysed in two logistic regression models in the first model we adjusted for all described co-variates, and in the second model we adjusted for white matter lesions and dementia. Analyses were studied separately in the later-born cohorts (born 1922 and 1930) and the earlier-born cohorts (born 1908, 1914, and 1918).

Finally, age adjusted logistic regressions analyzed the association between alcohol consumption, longstanding smoking, and frontal lobe atrophy. An interaction analysis was done to evaluate the interaction between smoking and alcohol consumption (longstanding smoking*more frequent alcohol consumption) in relation to frontal lobe atrophy (significance level $p < 0.20$). The sample was stratified by alcohol consumption (lower vs. higher), and we

For peer review only

re-analyzed the association between smoking and frontal lobe atrophy using logistic regressions within each strata. Correlations between the five outcome variables (cortical atrophy and white matter lesions) were tested and reported by Pearson correlation coefficient (95% CI), and the p values, of main findings, were multiplied with five (numbers of outcomes) to control for false positive result in multiple analyses. All analyses were performed with IBM SPSS version 24 statistical software.

Patient and public involvement

Patients and/or the public were not involved in the design, or conduct, or reporting, or dissemination plans of this research.

RESULTS

Characteristics of the study sample (n=369) are shown in Table 1. Mean age at the baseline examination 1968-69 was 44 years (SD ± 6 years) and cigarette smoking was reported by 32% (n=119) in 1968-69, 29% in 1974-75 and 25% in 1980-81. Sixteen percent in earlier-born cohorts (1908, 1914, and 1918), and 38% in the later-born cohorts (1922 and 1930), reported smoking in baseline (Table 2). Participants data from the CT-study in 2000-01 (mean age 76 years SD ± 6 years) are presented in Table 3. All outcome variables, atrophy and white matter lesions were associated to each other, according to Pearson correlation coefficient (see additional file).

Cigarette smoking in 1968-69 (adjusted OR 1.85; 95% CI 1.12-3.04), 1974-75 (OR 2.37; 95% CI 1.39-4.04) and 1980-81 (OR 2.47; 95% CI 1.41-4.33) was associated with late-life frontal lobe atrophy (Table 4). Report of smoking in the last examination (1980-81) was also associated with parietal lobe atrophy (OR 1.99; 95% CI 1.10-3.58). There was no association

For peer review only

between smoking and cortical atrophy in the temporal or occipital lobes, or with periventricular white matter lesions (Table 4).

Among the 351women who participated in all three midlife examinations, 65% were never-smokers, 12% reported smoking at one or two examinations, and 23% reported smoking at all three examinations (Table 5). The longstanding smokers (report of smoking in all 3 examinations) had higher prevalence of late-life frontal lobe atrophy compared to never-smokers (adjusted OR 2.63; 95% CI 1.44-4.78, p .003). The p value was further multiplied with five (numbers of outcomes) to control for false positive result in multiple analyses and were still significant (p .003 x 5= p .015), and the association also remained after additional adjusting for white matter lesions (OR 2.57, 95% CI 1.41-4.69). There were no associations between temporary smoking (smoking in 1-2 examination) and frontal lobe atrophy, nor between longstanding smoking and lesions in the other cortical and white matter areas (Table 5). The adjusted ORs (95% CI) between longstanding smoking and frontal lobe atrophy were 5.31 (0.59-47.84) in the earlier-born cohorts (born 1908, 1915 and 1918) and 2.49 (1.31-4.74) in the later-born cohorts (born 1922 and 1930).

There was an interaction between smoking and alcohol consumption (longstanding smoking*higher alcohol consumption) in relation to frontal lobe atrophy (age adjusted $p_{interaction}$ 0.13). The sample was thus stratified, to explore this interaction in more detail. In women with a more frequent alcohol consumption (>once weekly, n=101), longstanding smoking was associated with frontal lobe atrophy (adjusted OR 6.02, 95% CI 1.74-20.84), while no such association was found in those with low alcohol consumption (p > 0.05). In addition, a more frequent alcohol consumption was associated with longstanding smoking (p 0.02) but not with frontal lobe atrophy (p 0.63).

For peer review only

Seventeen women developed dementia (Alzheimer's disease or vascular dementia) during the study period between 1968 and 2001. The association between longstanding smoking and frontal lobe atrophy remained after additional adjustment for dementia (OR 2.50, 95% CI 1.38-4.56).

DISCUSSION

In this population-based sample of women followed over 32 years, we found that midlife tobacco smoking was associated with late-life frontal lobe atrophy. The strongest association was observed in those who reported smoking in all three midlife examinations and in women with a more frequent alcohol consumption. There was also an association between smoking in the last midlife examination (1980-81) and parietal lobe atrophy. Being a temporary smoker was not associated with late-life brain lesions. There were neither association between smoking and atrophy in other cortical areas or in the white matter. The findings remained after controlling for several potential confounders including lifestyle factors, vascular diseases, and dementia.

Our findings are difficult to compare with other studies, as few earlier studies have examined the effects of smoking on structural brain lesions on long follow-up periods of decades. Previous studies have mostly been conducted in cross-sectional samples of young and middle-aged people or were based on retrospective information on smoking habits. Among studies in younger populations, a meta-analysis of seven voxel-based morphometry (VBM) studies (n=418, median age 37) reported that chronic smokers had decreased gray matter volume in the anterior cingulate cortex,[12] i.e., the area surrounding the frontal part of corpus callosum.[27] Another meta-analysis, based on eleven VBM studies (n=1710, mean

For peer review only

age 42), established that smokers had smaller gray matter volumes in the prefrontal cortex and larger volumes in lingual cortex, compared to non-smokers.[13] In two recent published studies it was found that current smokers had smaller frontal and temporal gray matter volume (n = 698, mean age 50),[28] and thinning in the orbitofrontal cortex, insula, and entorhinal-middle temporal regions (n=82, mean age 45).[29] Taken together, less gray matter substance, in various brain regions, has been observed in chronic smokers, and most consistently in the prefrontal and frontal brain areas.

Several biological mechanisms may explain our findings regarding frontal lobe atrophy. Tobacco smoking affects the addiction-related regions in brain, [28, 30] and the frontal brain contains the highest densities of nicotinic acetylcholine receptors. [14] Nicotine interacts with the dopaminergic reward system in the forebrain,[15, 2] i.e., the mesolimbic system, [31, 32, 14] which connects the midbrain, with the basal ganglia, and the frontal cortex.[13] An acute nicotine intake causes activation of nicotinic receptors, while a chronic use can lead to desensitization of these receptors, due to the development of tolerance.[13] Therefore, a long-term activation and exhaustion of synapses might lead to receptor damage and neurodegeneration. [14] Data from animal models show that the neurotoxic effects of nicotine can lead to cell loss and synaptic alterations.[33, 34]. The fact that the mesolimbic pathway is part of the larger mesocortical pathway, might also explain the associations to parietal lobe atrophy,[15] which findings from this study support.

Interestingly, the association between smoking and frontal lobe atrophy varied depending on alcohol habits, i.e., women with a more frequent alcohol consumption had more frontal brain atrophy. Similar findings have been observed in earlier studies,[35] which suggests interaction effects between exposures linked to addiction behaviors.[15, 2] It is a well-known

For peer review only

fact that smoking co-occurs with higher alcohol consumption,[36] and findings from studies on alcohol disorders have shown an intricate interplay between smoking use and alcohol use.[29] Alcohol has also independently been associated with several negative brain outcomes, including smaller frontal volume and desensitization in the mesolimbic system.[8, 37]

We did not find an association between smoking and white matter lesions. Cigarette smoking gives exposure to carbon monoxide, free radicals and free oxygen species, which have negative cerebrovascular effects and increase the risks of arteriolosclerosis, ischemic demyelination, and lower blood flow.[28, 2, 16] The frontal cortex is vulnerable for vascular diseases, due to its reliance on multiple penetrating arteries in the underlying white matter, with few collaterals.[38, 39] When we used white matter lesions as a potential confounder between smoking and frontal lobe atrophy, the association between smoking and frontal lobe atrophy remained, i.e., this potential confounder did not modify the association. One explanation could be that the study only measured periventricular lesions on CT and not the total sub-cortical lesions volume, as there was no such information available. The findings are however in line with several other studies that neither found associations between smoking and less white matter volume.[28, 40, 41]

Frontal lobe shrinkage is frequently found in cognitive healthy older people and can occur without clinically correlated symptoms.[20-23] In our sample, over half of the population had some degree of frontal lobe atrophy. Only a minority of these (8%) were diagnosed with dementia and none of them with frontotemporal dementia. However, frontal atrophy can give rise to cognitive and psychiatric symptoms, which affect a persons' functioning, behavior,

For peer review only

and well-being.[42] Only a few studies have analyzed the association between smoking and frontotemporal dementia, and none of them have found evidence of causality.[43-45]

In our study, only those who reported smoking in all three midlife examinations had higher attendance of frontal lobe atrophy, while no associations were found in temporary smokers. Our findings suggest that the effect of smoking on frontal cortex decreases when smoking is stopped, as also has been shown for other adverse outcomes of smoking, such as cancer or cardiovascular disease.[46, 47] Another observation was that the risks (ORs) were slightly higher in smokers in the 1980-81 examination, compared to the 1968-69 and 1974-75 examinations, albeit not statistically significant. That can be a sign of an increased risk of smoking at higher ages, and that the women who smoked in 1980 mainly were the same as the longstanding smokers (i.e., 81 of 88 persons).

The strengths of our study are the population-based sampling method, the long follow-up period with a prospective design, repeated and detailed information on smoking habits during early and late midlife, and the possibility to control for several potential confounders. However, some methodological issues need to be considered. First, we had no detailed data on smoking habits before or after the study period, and no data on number of cigarettes/per day. Second, visual rating of brain atrophy and white matter lesions on CT is a rather crude method. CT-scans are less sensitive than MRI to detecting brain structural changes, but similar in measuring atrophy.[48] Third, attrition is a problem in long-term prospective studies. Compared to non-participants, participants in the CT-study were younger and had a lower mortality rate. They may thus be healthier and have less brain changes than the general population. If anything, this might likely have underestimated our findings. Fourth, we have no CT-data from the midlife examinations (1968-81) and can therefore not exclude that some

For peer review only

of the women might have had cortical atrophy already in midlife and that early subtle changes in the frontal lobes may e.g., led to addiction habits, such as smoking. Fifth, there could be a potential stigma related to the self-reporting of cigarettes and alcohol use. If a lower prevalence were reported, this might have underestimated our findings. Sixth, our study focused on smoking. Other forms of nicotine use, e.g., snuff or water pipe, was not measured. However, the prevalence of these latter habits should have been very low among middle-aged women between 1968 and 1981. Seventh, the sample size was sometimes too small for examining sub-groups, e.g., specific age cohorts. Finally, the study includes only Caucasian women. Thus, our results cannot be generalised to other populations.

Conclusion

The study found an association between tobacco smoking in midlife and frontal lobe atrophy in late life. The strongest association was found in women with a higher alcohol consumption. Only longstanding smoking was associated with higher risk of frontal atrophy, not sporadic smoking. Destruction of synapses and neurons in the frontal cortex might be a consequence of long-term toxic stimulation of the nicotine receptors. The findings have both clinical and public health implications, and further studies should analyse if longstanding tobacco use also are related to impaired cognitive functions.

Acknowledgements

The authors thank all members of the Prospective Population Study of Women group for their cooperation in data collection and management, and Valter Sundh for statistical assistance.

Contributorship statement

For peer review only

All authors (L.J., X.G., S.S., M.M.F., S.K., A.Z. and, I.S.) contributed to the conception or design of the work and drafted and approved the final version. All agreed to be accountable for all aspects of the work in ensuring that questions related to the accuracy or integrity of any part of the work are appropriately investigated and resolved.

Competing interests

None declared.

Funding

The study was financed by the ALF-agreement (ALF 716681), Swedish Research Council for Health, Working Life and Wellfare (2004-0145, 2006-0596, 2008-1111, 2010-0870, 2012-1138, 2013-1202, 2018-00471, AGECAP 2013-2300), Hjärnfonden (FO2014-0207, FO2016-0214, FO2018-0214, FO2019-0163), The Alzheimer's Association Stephanie B. Overstreet Scholars (IIRG-00-2159), The Alzheimer's Association Zenith Award (ZEN-01-3151), and Swedish Research Council (2005-8460, 2007-7462, 2012-5041, 2015-02830, 2019-01096, 2013-8717, NEAR 2017-00639).

Data sharing statement

Data are available upon reasonable request.

Ethics approval statement

This study received ethical approval by the Ethics Committee for Medical Research at the University of Gothenburg (Reference Ö402 99). All protocols were performed in accordance with the Declaration of Helsinki principles.

For peer review only

REFERENCES

1. Ekpu VU, Brown AK. The Economic Impact of Smoking and of Reducing Smoking Prevalence: Review of Evidence. *Tob Use Insights* 2015;8,1-35.doi:10.4137/TUI.S15628

2. Swan GE, Lessov-Schlaggar CN The effects of tobacco smoke and nicotine on cognition and the brain. *Neuropsychol Rev* 2007;17(3),259-273.doi:10.1007/s11065-007-9035-9

3. Johansson L, Skoog I, Gustafson DR et al..Midlife psychological distress associated with late-life brain atrophy and white matter lesions: a 32-year population study of women. *Psychosom Med* 2012;74(2),120-125.doi:PSY.0b013e318246eb10

4. Pini L, Pievani M, Bocchetta M et al.. Brain atrophy in Alzheimer's Disease and aging. *Ageing Res Rev* 2016;30,25-48.doi:10.1016/j.arr.2016.01.002

5. Pantoni L. Cerebral small vessel disease: from pathogenesis and clinical characteristics to therapeutic challenges. *Lancet Neurol* 2010;9(7),689-701.doi:10.1016/S1474-4422(10)70104-6

6. Agosta F, Vossel KA, Miller BL, et al.. Apolipoprotein E epsilon4 is associated with disease-specific effects on brain atrophy in Alzheimer's disease and frontotemporal dementia. *Proc Natl Acad Sci U S A* 2009;106(6), 2018-2022.doi:10.1073/pnas.0812697106

7. Gazdzinski S, Kornak J, Weiner MW et al.. Body mass index and magnetic resonance markers of brain integrity in adults. *Ann Neurol* 2008;63(5),652-657.doi:10.1002/ana.21377

For peer review only

8. Kubota M, Nakazaki S, Hirai S et al.. Alcohol consumption and frontal lobe shrinkage: study of 1432 non-alcoholic subjects. *J Neurol Neurosurg Psychiatry*2001;71(1), 104-106. Retrieved from https://www.ncbi.nlm.nih.gov/pubmed/11413273

9. Lou B, Chen M, Luo X et al.. Reduced right frontal fractional anisotropy correlated with early elevated plasma LDL levels in obese young adults. *PLoS One* 2014;9(10),e108180.doi:10.1371/journal.pone.0108180

10. Zhang Y, Zhang X, Zhang J et al.. Gray matter volume abnormalities in type 2 diabetes mellitus with and without mild cognitive impairment. *Neurosci Lett* 2014;562,1-6.doi:10.1016/j.neulet.2014.01.006

11. Gray JC, Thompson M, Bachman C et al.. Associations of cigarette smoking with gray and white matter in the UK Biobank. *Neuropsychopharmacology* 2020;45(7):1215-1222. doi: 10.1038/s41386-020-0630-2

12. Pan P, Shi H, Zhong J et al.. Chronic smoking and brain gray matter changes: evidence from meta-analysis of voxel-based morphometry studies. *Neurol Sci* 2013;34(6),813-817.doi:10.1007/s10072-012-1256-x

13. Zhong J, Shi H, Shen Y et al.. Voxelwise meta-analysis of gray matter anomalies in chronic cigarette smokers. *Behav Brain Res* 2016;311,39-45.doi:10.1016/j.bbr.2016.05.016

For peer review only

14. Kober H, Deleone CM. Smoking and Neuroimaging: A Review. *Curr Cardiovasc Risk Rep* 2011;5(6),484-491.doi:10.1007/s12170-011-0201-5

15. Jasinska AJ, Zorick T, Brody AL et al.. Dual role of nicotine in addiction and cognition: a review of neuroimaging studies in humans. *Neuropharmacology* 2014;84,111-122.doi:10.1016/j.neuropharm.2013.02.015

16. Tomonaga M, Yamanouchi H, Tohgi H et al.. Clinicopathologic study of progressive subcortical vascular encephalopathy (Binswanger type) in the elderly. *J Am Geriatr Soc* 1982;30(8),524-529.

17. Durazzo TC, Meyerhoff DJ. Neurobiological and neurocognitive effects of chronic cigarette smoking and alcoholism. *Front Biosci* 2007;12,4079-4100.doi:10.2741/2373

18. Bengtsson C, Blohme G, Hallberg L, et al.. The study of women in Gothenburg 1968-1969--a population study. General design, purpose and sampling results. *Acta Med Scand* 1973;193(4), 311-318.

19. Guo X, Skoog I, Matousek M et al.. A population-based study on motor performance and white matter lesions in older women. *J Am Geriatr Soc* 2000;48(8),967-970.

20. Simoni M, Pantoni L, Pracucci G et al.. Prevalence of CT-detected cerebral abnormalities in an elderly Swedish population sample. *Acta Neurol Scand* 2008;118(4),260-267. doi:ANE1010

For peer review only

21. De Leon MJ, Ferris SH, George AE, et al.. Computed tomography evaluations of brain-behavior relationships in senile dementia of the Alzheimer's type. *Neurobiology of Aging* 1980;1(1),69-79.

22. Thompson PM, Hayashi KM, de Zubicaray G et al.. Dynamics of gray matter loss in Alzheimer's disease. *J Neurosci* 2003;33(3),994-1005.doi:23/3/994 [pii]

23. Gustafson D, Lissner L, Bengtsson C et al.. A 24-year follow-up of body mass index and cerebral atrophy. *Neurology* 2004;63(10),1876-1881.doi:63/10/1876[pii]

24. Skoog I, Palmertz B, Andreasson LA The prevalence of white-matter lesions on computed tomography of the brain in demented and nondemented 85-year-olds. *J Geriatr Psychiatry Neurol* 1994;7(3),169-175

25. Guo X, Waern M, Sjogren K et al.. Midlife respiratory function and Incidence of Alzheimer's disease: a 29-year longitudinal study in women. *Neurobiology of Aging* 2007;28(3),343-350.doi:S0197-4580(06)00041-8

26. Skoog I, Nilsson L, Palmertz B et al.. A population-based study of dementia in 85-year-olds. *N Engl J Med* 1993;328(3),153-158.doi:10.1056/NEJM199301213280301

27. Taylor SF, Martis B, Fitzgerald KD et al.. Medial frontal cortex activity and loss-related responses to errors. *J Neurosci* 2006;26(15),4063-4070.doi:10.1523/JNEUROSCI.4709-05.2006

For peer review only

28. Elbejjani M., Auer R, Jacobs DR et al..Cigarette smoking and gray matter brain volumes in middle age adults: the CARDIA Brain MRI sub-study. *Transl Psychiatry* 2019;9(1),78.doi:10.1038/s41398-019-0401-1

29. Durazzo TC, Meyerhoff DJ, Yoder KK. Cigarette smoking is associated with cortical thinning in anterior frontal regions, insula and regions showing atrophy in early Alzheimer's Disease. *Drug Alcohol Depend* 2018;192, 277-284.doi:10.1016/j.drugalcdep.2018.08.009

30. Wang K, Yang J, Zhang S et al.. The neural mechanisms underlying the acute effect of cigarette smoking on chronic smokers. *PLoS One* 2014;9(7),e102828.doi:10.1371/journal.pone.0102828

31. Dinur-Klein L, Dannon P, Hadar A, et al.. Smoking cessation induced by deep repetitive transcranial magnetic stimulation of the prefrontal and insular cortices: a prospective, randomized controlled trial. *Biol Psychiatry* 2014;76(9),742-749.doi:10.1016/j.biopsych.2014.05.020

32. Hartwell KJ, Prisciandaro JJ, Borckardt J et al.. Real-time fMRI in the treatment of nicotine dependence: a conceptual review and pilot studies. *Psychol Addict Behav* 2013;27(2),501-509.doi:10.1037/a0028215

33. Chen WJ, Edwards RB, Romero RD, et al.. Long-term nicotine exposure reduces Purkinje cell number in the adult rat cerebellar vermis. *Neurotoxicol Teratol* 2003; 25(3), 329-334. doi:10.1016/s0892-0362(02)00350-1

For peer review only

34. Trauth JA, McCook EC, Seidler FJ Modeling adolescent nicotine exposure: effects on cholinergic systems in rat brain regions. *Brain Res* 2000;873(1),18-25.doi:10.1016/s0006-8993(00)02465-3

35. Elbejjani M, Auer R, Dolui, S et al.. Cigarette smoking and cerebral blood flow in a cohort of middle-aged adults. *J Cereb Blood Flow Metab* 2019;39(7), 1247-1257.doi:10.1177/0271678X18754973

36. Room R Smoking and drinking as complementary behaviours. *Biomed Pharmacother* 2004;58(2),111-115.doi:10.1016/j.biopha.2003.12.003

37. Rando K, Hong KI, Bhagwagar Z et al.. Association of frontal and posterior cortical gray matter volume with time to alcohol relapse: a prospective study. *Am J Psychiatry* 2011;168(2),183-192.doi:10.1176/appi.ajp.2010.10020233

38. Chen Y, Chen X, Xiao W, et al.. Frontal lobe atrophy is associated with small vessel disease in ischemic stroke patients. *Clin Neurol Neurosurg* 2009;111(10), 852-857.doi:10.1016/j.clineuro.2009.08.019

39. Foster V, Oakley AE, Slade JY et al.. Pyramidal neurons of the prefrontal cortex in post-stroke, vascular and other ageing-related dementias. *Brain* 2014;137(Pt 9),2509-2521.doi:10.1093/brain/awu172

For peer review only

40. Fritz HC, Wittfeld K, Schmidt CO et al.. Current smoking and reduced gray matter volume-a voxel-based morphometry study. *Neuropsychopharmacology* 2014;39(11),2594-2600.doi:10.1038/npp.2014.112

41. Gallinat J, Meisenzahl E, Jacobsen LK et al.. Smoking and structural brain deficits: a volumetric MR investigation. *Eur J Neurosci* 2006;24(6),1744-1750.doi:10.1111/j.1460-9568.2006.05050.x

42. Chan DK, Reutens S, Liu DK, et al.. Frontotemporal dementia - features, diagnosis and management. *Aust Fam Physician* 2011;40(12),968-972

43. Kalkonde YV, Jawaid A, Qureshi S et al.. Medical and environmental risk factors associated with frontotemporal dementia: a case-control study in a veteran population. *Alzheimers Dement* 2012;8(3),204-210.doi:10.1016/j.jalz.2011.03.011

44. Rasmussen Eid H, Rosness TA, Bosnes O et al.. Smoking and Obesity as Risk Factors in Frontotemporal Dementia and Alzheimer's Disease: The HUNT Study. *Dement Geriatr Cogn Dis Extra* 2019;9(1),1-10.doi:10.1159/000495607

45. Tremolizzo L, Bianchi E, Susani E et al.. Voluptuary Habits and Risk of Frontotemporal Dementia: A Case Control Retrospective Study. *J Alzheimers Dis* 2017;60(2),335-340.doi:10.3233/JAD-170260

For peer review only

46. Lee H, Son YJ. Influence of Smoking Status on Risk of Incident Heart Failure: A Systematic Review and Meta-Analysis of Prospective Cohort Studies. *Int J Environ Res Public Health* 2009;16(15).doi:10.3390/ijerph16152697

47. Parsons A, Daley A, Begh R et al.. Influence of smoking cessation after diagnosis of early stage lung cancer on prognosis: systematic review of observational studies with meta-analysis. *BMJ* 2010;340,b5569.doi:10.1136/bmj.b5569

48. Frisoni GB. Structural imaging in the clinical diagnosis of Alzheimer's disease: problems and tools. *J Neurol Neurosurg Psychiatry* 2001;70(6),711-718.

For peer review only

Table 1 Characteristics of the study sample (n=369)

Baseline age (years), mean ± SD	43.8 ± 5.6
Birth year 1908, n (%)	2 (0.5)
Birth year 1914, n (%)	19 (5.1)
Birth year 1918, n (%)	80 (21.7)
Birth year 1922, n (%)	105 (28.5)
Birth year 1930, n (%)	163 (44.2)
Current smoking, n (%)	
Examination 1968-68	119 (32.2)
Examination 1974-75	104 (28.8)
Examination 1980-81	88 (25.0)
Co-variates	
Alcohol consumption (> once weekly) [a], n (%)	101 (27.4)
Body mass index (BMI) [ad], mean ± SD	23.46 ± 0.17
Cardio- and cerebrovascular diseases (CCVD)[e], n (%)	53 (14.4)
Cholesterol (mmol/l) [ad], mean ± SD	6.59 ± 0.08
Hypertension [ac], n (%)	77 (20.9)
Psychological stress [ab], n (%)	67 (18.2)
Low physical activity (< 4 hour/week) [a], n (%)	51 (13.8)
Dementia[e], n (%)	17 (4.6)

[a] Measured at baseline, 1968

[b] 8 with missing information

[c] 17 with missing information

[d] 1 with missing information

[e] Measured until December 31, 2002

For peer review only

For peer review only - http://bmjopen.bmj.com/site/about/guidelines.xhtml

Table 2 Prevalence of cigarette smoking in midlife examinations, presented by birth cohort

	Born 1908	Born 1914	Born 1918	Born 1922	Born 1930
Current smoking 1968-69, n (%)	0 (0)	2 (10.5)	16 (20.0)	38 (36.2)	63 (38.7)
Currant smoking 1974-75, n (%)	0 (0)	2 (10.5)	14 (18.2)	28 (26.9)	60 (37.7)
Current smoking 1980-81, n (%)	0 (0)	2 (10.5)	11 (14.9)	23 (22.8)	52 (33.3)

For peer review only

Table 3 Prevalence of cortical atrophy and white matter lesions on brain CT-scans in 2000-01, presented by birth cohort

	Born 1908 [a] (n=2)	Born 1914 [b] (n=19)	Born 1918 [c] (n=80)	Born 1922 [d] (n=105)	Born 1930 [e] (n=163)	Total sample (n=369)
Temporal lobe atrophy, n (%)	2 (100)	17 (89.5)	57 (71.3)	57 (54.3)	52 (31.9)	185 (50.1)
Frontal lobe atrophy, n (%)	1 (50)	17 (89.5)	57 (71.3)	62 (59.0)	60 (36.8)	197 (53.4)
Parietal lobe atrophy, n (%)	1 (50)	15 (78.9)	42 (52.5)	49 (46.7)	27 (16.6)	134 (36.3)
Occipital lobe atrophy, n (%)	1 (50)	12 (63.2)	33 (41.3)	27 (25.7)	10 (6.1)	83 (22.5)
White matter lesions, n (%)	2 (100)	15 (78.9)	56 (70.0)	73 (69.5)	66 (40.5)	212 (57.5)

CT: Computerized tomography

[a] Aged 92 to 93 years; [b] Aged 86 to 87 years; [c] Aged 82 to 83 years; [d] Aged 78 to 79 years; [e] Aged 70 to 71 years

For peer review only

Table 4 Associations between midlife smoking and late-life structural brain changes on CT-scans in 2000

	Tobacco smoking in 1968 (n=119)	Tobacco smoking in 1974 (n=104)	Tobacco smoking in 1980 (n=88)
Temporal lobe atrophy			
No. of cases	53	50	45
OR_1 (95% CI)	0.93 (0.58-1.49)	1.23 (0.75-2.03)	1.53 (0.90-2.61)
OR_2 (95% CI)	0.95 (0.58-1.56)	1.27 (0.75-2.13)	1.60 (0.92-2.79)
Frontal lobe atrophy			
No. of cases	69	63	54
OR_1 (95% CI)	**1.80 (1.11-2.92)**	**2.24 (1.34-3.73)**	**2.30 (1.34-3.95)**
OR_2 (95% CI)	**1.85 (1.12-3.04)**	**2.37 (1.39-4.04)**	**2.47 (1.41-4.33)**
Parietal lobe atrophy			
No. of cases	42	36	34
OR_1 (95% CI)	1.32 (0.80-2.19)	1.37 (0.81-2.34)	**1.89 (1.07-3.33)**
OR_2 (95% CI)	1.38 (0.81-2.34)	1.37 (0.79-2.39)	**1.99 (1.10-3.58)**
Occipital lobe atrophy			
No. of cases	24	21	20
OR_1 (95% CI)	1.21 (0.67-2.20)	1.35 (0.73-2.55)	1.75 (0.90-3.39)
OR_2 (95% CI)	1.25 (0.67-2.32)	1.35 (0.70-2.61)	1.84 (0.92-3.67)
White matter lesions			
No. of cases	68	61	52
OR_1 (95% CI)	1.26 (0.79-2.02)	1.41 (0.86-2.32)	1.51 (0.89-2.56)
OR_2 (95% CI)	1.43 (0.74-2.75)	1.83 (0.94-3.57)	1.27 (0.62-2.64)

Logistic regression analyses; OR (95%): odds ratio (95% confidence interval)

CT: Computerized tomography

OR_1 adjusted for age

OR_2 adjusted for age, alcohol consumption, BMI, CCVD, cholesterol, hypertension, physical activity, and psychological stress

For peer review only

Table 5 Associations between midlife smoking in multiple examinations, between 1968-80, and late-life structural brain changes on CT-scans in 2000 (n=351)

	No tobacco smoking in any examination (n=227)	Tobacco smoking in 1-2 examination (n=43)	Tobacco smoking in 3 examinations (n=81)
Temporal lobe atrophy<br>No. of cases	121	17	40
OR_1 (95% CI)	1.0 (ref.)	0.74 (0.37-1.51)	1.24 (0.71-2.17)
OR_2 (95% CI)	1.0 (ref.)	0.76 (0.37-1.56)	1.28 (0.72-2.30)
Frontal lobe atrophy<br>No. of cases	118	19	51
OR_1 (95% CI)	1.0 (ref.)	0.96 (0.48-1.91)	**2.36 (1.36-4.18)**
OR_2 (95% CI)	1.0 (ref.)	1.00 (0.49-2.04)	**2.63 (1.44-4.78)**
Parietal lobe atrophy<br>No. of cases	84	12	31
OR_1 (95% CI)	1.0 (ref.)	0.94 (0.44-2.01)	1.69 (0.94-3.03)
OR_2 (95% CI)	1.0 (ref.)	0.99 (0.45-2.18)	1.82 (0.98-3.39)
Occipital lobe atrophy<br>No. of cases	55	7	18
OR_1 (95% CI)	1.0 (ref.)	0.99 (0.39-2.50)	1.57 (0.79-3.12)
OR_2 (95% CI)	1.0 (ref.)	0.96 (0.37-2.46)	1.60 (0.77-3.32)
White matter lesions<br>No. of cases	131	22	49
OR_1 (95% CI)	1.0 (ref.)	0.97 (0.49-1.92)	1.55 (0.89-2.69)
OR_2 (95% CI)	1.0 (ref.)	1.44 (0.56-3.72)	1.53 (0.71-3.29)

Logistic regression analyses; OR (95%): odds ratio (95% confidence interval)

CT: Computerized tomography

OR_1 adjusted for age

OR_2 adjusted for age, alcohol consumption, BMI, CCVD, cholesterol, hypertension, physical activity, and psychological stress

For peer review only

Additional file Correlation matrix presenting Pearson correlation coefficient (95% CI) between the five outcome variables

	Temporal lobe atrophy	Frontal lobe atrophy	Parietal lobe atrophy	Occipital lobe atrophy
Frontal lobe atrophy	0.49 (0.41-0.57) ***			
Parietal lobe atrophy	0.52 (0.44-0.59) ***	0.55 (0.47-0.62) ***		
Occipital lobe atrophy	0.54 (0.46-0.61) ***	0.48 (0.39-0.55) ***	0.70 (0.64-0.75) ***	
White matter lesions	0.23 (0.13-0.32) ***	0.15 (0.05-0.25) **	0.15 (0.05-0.25) **	0.21 (0.12-0.31) ***

The Pearson correlation coefficient (95% CI), *** = p value <0.001, ** = p value <0.01

For peer review only

STROBE Statement—Checklist of items that should be included in reports of ***cohort studies***

	Item No	Recommendation	Page No
**Title and abstract**	1	(*a*) Indicate the study's design with a commonly used term in the title or the abstract<br>(*b*) Provide in the abstract an informative and balanced summary of what was done and what was found	1
**Introduction**			
Background/rationale	2	Explain the scientific background and rationale for the investigation being reported	2,3,4
Objectives	3	State specific objectives, including any prespecified hypotheses	2,3,4
**Methods**			
Study design	4	Present key elements of study design early in the paper	4,5,6
Setting	5	Describe the setting, locations, and relevant dates, including periods of recruitment, exposure, follow-up, and data collection	4,5,6
Participants	6	(*a*) Give the eligibility criteria, and the sources and methods of selection of participants. Describe methods of follow-up<br>(*b*) For matched studies, give matching criteria and number of exposed and unexposed	4,5,6
Variables	7	Clearly define all outcomes, exposures, predictors, potential confounders, and effect modifiers. Give diagnostic criteria, if applicable	4-7
Data sources/ measurement	8*	For each variable of interest, give sources of data and details of methods of assessment (measurement). Describe comparability of assessment methods if there is more than one group	4-7
Bias	9	Describe any efforts to address potential sources of bias	4-7, 11
Study size	10	Explain how the study size was arrived at	4-5
Quantitative variables	11	Explain how quantitative variables were handled in the analyses. If applicable, describe which groupings were chosen and why	4-7
Statistical methods	12	(*a*) Describe all statistical methods, including those used to control for confounding<br>(*b*) Describe any methods used to examine subgroups and interactions<br>(*c*) Explain how missing data were addressed<br>(*d*) If applicable, explain how loss to follow-up was addressed<br>(*e*) Describe any sensitivity analyses	7
**Results**			
Participants	13*	(a) Report numbers of individuals at each stage of study—eg numbers potentially eligible, examined for eligibility, confirmed eligible, included in the study, completing follow-up, and analysed<br>(b) Give reasons for non-participation at each stage<br>(c) Consider use of a flow diagram	7,8
Descriptive data	14*	(a) Give characteristics of study participants (eg demographic, clinical, social) and information on exposures and potential confounders<br>(b) Indicate number of participants with missing data for each variable of interest<br>(c) Summarise follow-up time (eg, average and total amount)	7,8
Outcome data	15*	Report numbers of outcome events or summary measures over time	7,8

For peer review only - http://bmjopen.bmj.com/site/about/guidelines.xhtml

Main results	16	(*a*) Give unadjusted estimates and, if applicable, confounder-adjusted estimates and their precision (eg, 95% confidence interval). Make clear which confounders were adjusted for and why they were included<br>(*b*) Report category boundaries when continuous variables were categorized<br>(*c*) If relevant, consider translating estimates of relative risk into absolute risk for a meaningful time period	7-9
Other analyses	17	Report other analyses done—eg analyses of subgroups and interactions, and sensitivity analyses	7,8
**Discussion**			
Key results	18	Summarise key results with reference to study objectives	8-12
Limitations	19	Discuss limitations of the study, taking into account sources of potential bias or imprecision. Discuss both direction and magnitude of any potential bias	12
Interpretation	20	Give a cautious overall interpretation of results considering objectives, limitations, multiplicity of analyses, results from similar studies, and other relevant evidence	12
Generalisability	21	Discuss the generalisability (external validity) of the study results	12
**Other information**			
Funding	22	Give the source of funding and the role of the funders for the present study and, if applicable, for the original study on which the present article is based	12

*Give information separately for exposed and unexposed groups.

Note: An Explanation and Elaboration article discusses each checklist item and gives methodological background and published examples of transparent reporting. The STROBE checklist is best used in conjunction with this article (freely available on the Web sites of PLoS Medicine at http://www.plosmedicine.org/, Annals of Internal Medicine at http://www.annals.org/, and Epidemiology at http://www.epidem.com/). Information on the STROBE Initiative is available at http://www.strobe-statement.org.

For peer review only
